# NSG Mice Facilitate *ex vivo* Characterization of Ewing Sarcoma Lung Metastasis Using the PuMA Model

**DOI:** 10.3389/fonc.2021.645757

**Published:** 2021-03-22

**Authors:** Renata Scopim-Ribeiro, Michael M. Lizardo, Hai-Feng Zhang, Anne-Chloé Dhez, Chistopher S. Hughes, Poul H. Sorensen

**Affiliations:** ^1^Department of Molecular Oncology, BC Cancer, Vancouver, BC, Canada; ^2^Department of Pathology & Laboratory Medicine, University of British Columbia, Vancouver, BC, Canada

**Keywords:** lung metastasis, Ewing sarcoma, osteosarcoma, PuMA, NSG mice, NK cells

## Abstract

Ewing sarcoma (EwS) is a highly malignant bone and soft tissue tumor primarily affecting children and young adults. While most patients initially respond well to conventional front-line therapy, frequent metastasis results in poor 5-year overall survival rates for this disease. Accordingly, there is a critical need to develop better models to understand EwS metastasis. We and others previously used the *ex vivo* pulmonary metastasis assay (PuMA) to study lung metastasis in solid tumors including osteosarcoma (OS), but this technique has to date not been achievable for EwS. PuMA involves tail vein injection of fluorescent tumor cells into NOD-SCID mice, followed by their visualization in long-term cultures of tumor-bearing lung explants. Here we demonstrate successful implementation of PuMA for EwS cells using NOD-SCID-IL2 receptor gamma null (NSG) immunocompromised mice, which demonstrated high engraftment of EwS cell lines compared to NOD-SCID mice. This may be linked to immune permissiveness required by EwS cells, as increased basal cytotoxicity of EwS cells was observed in NOD-SCID compared to NSG lung sections, possibly due to the absence of natural killer (NK) cell activity in the latter. Together, our data demonstrate the utility of NSG mice for PuMA modeling of EwS lung metastasis.

## Introduction

EwS is characterized in the majority of cases by the expression of EWSR1-FLI1 or EWSR1-ERG fusion proteins (1, 2). These oncoproteins function as chimeric transcription factors to regulate a broad range of candidate genes, leading to characteristic signatures of expression for these tumors (3–5). EwS tumors are highly metastatic, with dissemination occurring most commonly to lungs, bones, or bone marrow, with 20% of patients presenting with circulating EwS tumor cells at diagnosis (6). The 5-year overall survival of patients with metastasis at diagnosis or with recurrent disease remains dismal (7), highlighting the pressing need for extensive study into the mechanisms regulating EwS metastasis toward potential development of preventative therapeutic treatments. To facilitate the rapid progression of research into EwS metastasis, the development of cutting-edge methods that enable robust examination of *in vivo* tumorigenic behavior is essential.

The PuMA system has been used over the past 10 years to study tumor cell lung colonization, particularly OS (8–10). In PuMA experiments, green fluorescent protein (GFP), or other fluorescently-labeled tumor cells are injected via the tail vein into NOD-SCID mice. Growth of tumor cells is then monitored using fluorescence microscopy of lung sections that are cultured as *ex vivo* explants for several weeks. As such, PuMA recapitulates the arrest of tumor cells in the lung, and furthermore, allows for the direct quantification of metastatic cell growth in living lung tissues. Another major advantage over conventional metastasis models is that these studies can be performed without the need for whole animal studies, allowing for short-term temporal comparison of anti-metastatic effects of given interventions (e.g., candidate gene knockdown or specific drug treatments) in a 3D microenvironment (11–13).

The PuMA system has conventionally used the NOD-SCID immunocompromised mouse as the basis for *ex vivo* lung colonization studies (8–10). The NOD-scid IL2rγ^null^ (NSG) mouse is a variant of NOD-SCID mice and was generated by introducing an X-linked IL2 receptor common gamma chain mutation to the NOD/Lt strain background carrying the SCID mutation (14). The interleukin 2 receptor gamma (IL2RG) gene produces the common gamma chain subunit, which serves as the signaling subunit for multiple cytokines (15). NOD-SCID and NSG mouse models share T and B cell depletion, loss of C5 complement, and impaired innate immunity (16). However, due to the absence of functional receptors for IL-2 and other cytokines, NSG mice are also deficient in NK cells (14). The NSG model has successfully been used to study metastasis of numerous different cancer types (16–18).

Despite the demonstrated utility of this experimental approach to the study of metastatic disease in OS, EwS cell lines have historically failed to grow in PuMA. In the present study, we set out to determine if alternative culture methods and a different mouse strain, namely NSG, would provide a lung microenvironment that is permissive for studying EwS metastatic growth in an adapted PuMA model. Our results demonstrate that PneumaCult™-ALI (PC medium) supports EwS cell survival and the maintenance of proper lung architecture in *ex vivo* cultures, in contrast to the B medium formulation used for conventional PuMA studies. In addition, we observe that in contrast to standard NOD-SCID mice, NSG mice are necessary for propagating EwS cells in the PuMA system. From these results we observe that enhanced immune permissiveness in NSG mice lacking NK cell activity may facilitate growth of EwS cells in the PuMA model compared to NOD-SCID mice, which have residual NK cells. Together, the results of this work demonstrate the novel adaptation of PuMA toward facilitating the critical study of metastatic disease in EwS.

## Materials and Methods

### Cell Lines and Reagents

The NK92 cell line was kindly provided by Dr. Dixie Mager. A673 and TC32 cells were purchased from ATCC and the plasmid for tdTomato stable expression was provided by Dr. John Ronald (Western University, Canada). eGFP-expressing MG63.3 cells were established by Ren et al. (9) and provided by Dr. Rosandra Kaplan (Pediatric Oncology Branch, National Cancer Institute). All cell lines were tested for mycoplasma on a regular basis using the LookOut Mycoplasma Detection Kit (Sigma).

### PuMA

Studies were conducted under the UBC animal care certificate #A19-0143. PuMA assays were performed as described previously (8), with several modifications. Specifically, 1 × 10^6^ viable A673 or TC32 (tdTomato^+^) cells were injected via tail-veins into either NOD-SCID or NSG 5–8 week-old female mice. Within 15–20 min of injection, mice were euthanized according to institutional animal care guidelines. A 1.2% agarose solution diluted 1:1 (v/v) in PneumaCult™-ALI (PC) basal media (STEMCELL technologies #05002) with 10x supplement (#05003) culture medium at 37°C was used to insufflate the lung via cannulation of the trachea with a Surflo Teflon IV Catheter 20G × 1.25” (Terumo Medical Canada Inc.). Lungs were then removed and placed in an ice-cold solution of PBS containing 100 U/mL penicillin and 100 μg/mL streptomycin and incubated at 4°C for 20-min. Transverse sections (1–2 mm in thickness) were made from each lobe and placed on a single 1.5 × 0.7-cm sterile Gelfoam (Pfizer) section pre-incubated overnight in a 6-well-plate with PC medium. For continuous culture, lung sections were incubated at 37°C in a humidified environment with 5% CO_2_. Every 48 h, culture medium was replaced with fresh PC.

### Renal Subcapsular Implantation Model

Studies were conducted under the UBC animal care certificate #A19-0143. In brief, as described by Mendoza-Naranjo et al. (19), xenograft cell blocks for implantation were prepared using 1 × 10^6^ viable TC32 EwS cells (tdTomato^+^) and implanted under the renal capsules of 6–8 week-old NSG immunocompromised male mice from the Animal Resource Center in the BC Cancer Research Center (*n* = 4). Animals were maintained according to UBC Animal Care Committee (ACC) regulations. Five weeks post-inoculation, mice were euthanized and lungs were collected for analysis.

### Histology/Immunohistochemistry

Paraffin-embedded lung sections were sectioned and processed for standard H&E's or immunohistochemistry (IHC) using antibodies to CD99 (Abcam #ab27271) followed by ImmunoHistoMount (ImmunoBioscience #AR-6503-01) staining. Immunofluorescence was performed using primary mouse anti-CD99 antibody [HO36-1.1] (Abcam #ab212605) and anti-Granzyme B antibody [EPR22645-206] (Abcam #ab255598). Secondary anti-mouse Alexa 594 (Thermo Scientific #A11005) and anti-rabbit Alexa 488 (Thermo Scientific #A11008) antibodies were used for detection. Images were acquired using Zeiss LSM 800 Airyscan system controlled with Zen Blue software (version 2.6).

### Fluorescent Imaging

Fluorescent images were acquired using a Colibri Observer Z1 microscope equipped with an Axiocam MRm and controlled using Zen Blue Software (version 3.1). Areas of fluorescence as a measure of metastatic burden were calculated using ImageJ and expressed as means of the fluorescent area in % area of each lung section across 10 individual sections. Fluorescent areas were normalized to day 0 and expressed as fold-changes. The Zeiss LSM 800 Airyscan system was used for confocal imaging using Zen Blue software (version 2.6).

### *In vitro* NK Cytotoxicity Assay

NK92 cell viability was determined by Trypan blue (Sigma-Aldrich) and maintained at 91–93% across experiments. NK92 effector cells were added 24 h after A673, TC32, or MG63.3 target cell seeding at the indicated effector to target (E:T) ratios. After 24 h in co-culture, target cells were fixed and stained with crystal violet. Absorbance was quantified using SpectraMax i3 plate reader (Molecular Devices).

## Results

**Figure 1:**
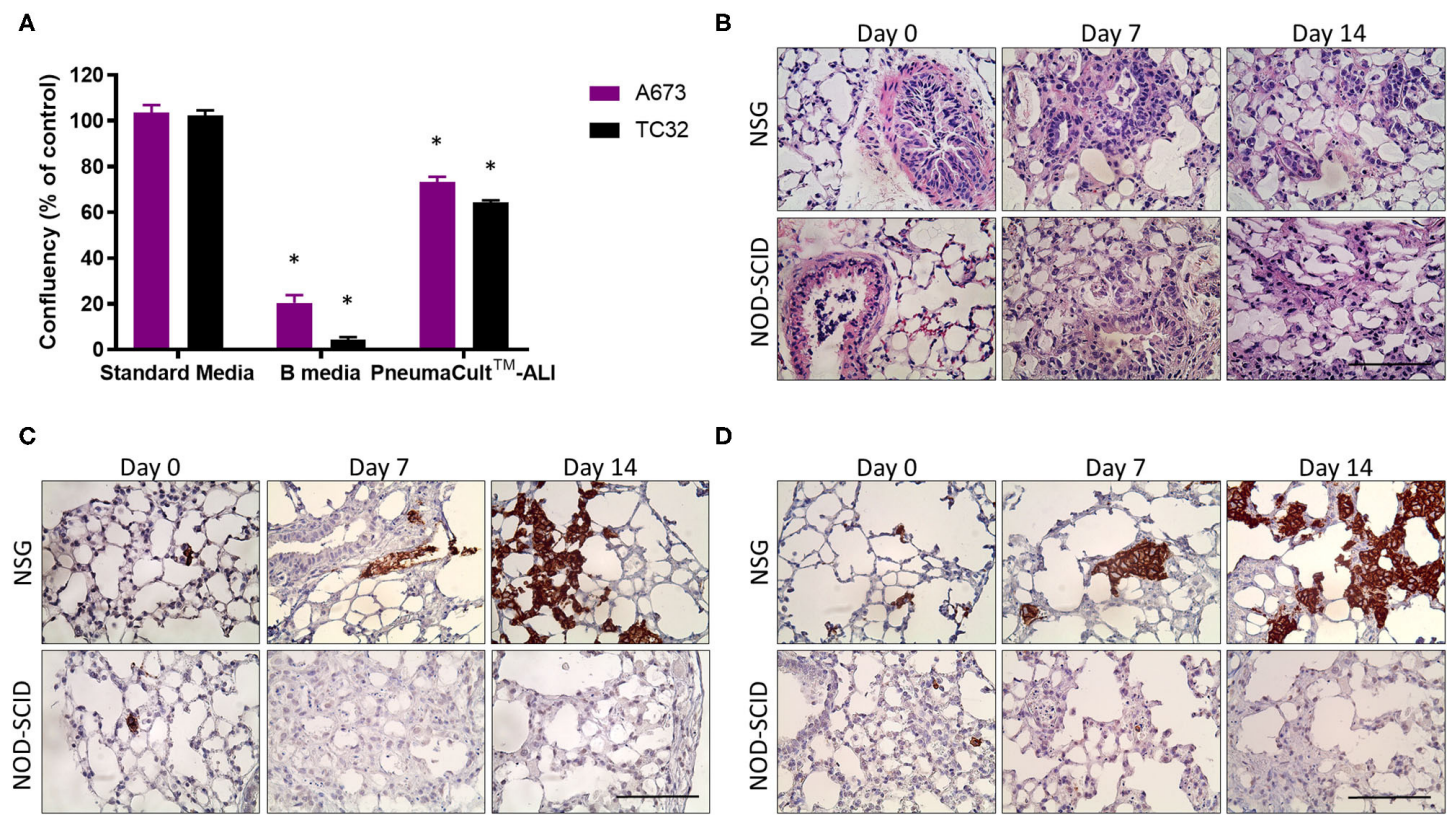
PneumaCult™-ALI [PC] medium maintains lung architecture during *ex vivo* growth. **(A)** Percentage of viable EwS cells cultured in B- and PC-medium relative to standard culture conditions for 96 h. Values reported represent the mean ± standard deviation (SD) of at least three independent experiments. Statistical analysis of differences between pairs was performed using a two-way ANOVA with a Tukey's *post-hoc* test (*p < 0.01). **(B)** H&E sections of NOD-SCID and NSG mouse lungs showing integrity of lung structures after 7 and 14 days. **(C)** Immunohistochemistry showing CD99 immunoreactive A673 and **(D)** TC32 cells in NOD-SCID and NSG mice lung slices at 0, 7, and 14 days post-injection. Three animals were included per group (*n* = 3) and a minimum of 10 lung tissue sections were analyzed per animal. Scale bars = 100 μm.

While current *in vivo* xenograft models of EwS are effective for analyzing primary tumor growth, they often fail to allow assessment of metastatic disease before experimental endpoints are reached. The PuMA method facilitates the quantification and characterization of metastatic lesions in lung tissues over 14 days or more in a pathophysiologic setting, where interactions with lung epithelial cells and stromal elements are retained (8–10). PuMA has historically used NOD-SCID mice and so-called B medium for culturing lung explants, but we have observed these conditions to be incompatible with EwS cell growth in the PuMA model. We wondered whether this might be due to toxicity of EwS cells when cultured in B media, or the use of NOD-SCID mouse that have residual NK cell activity. We first compared EwS cell viability in B medium vs. a commercially available formulation called PneumaCult™-ALI (PC) medium designed for human airway epithelial cells cultured at the air-liquid interface (20). Using crystal violet staining, we observed that in contrast to B medium, EwS cells survived in PC medium (Figure 1A). Culture in PC medium was also observed to maintain pulmonary microarchitecture of both NOD-SCID and NSG mice over 14 days of PuMA *ex vivo* cultivation (Figure 1B).

**Figure 2:**
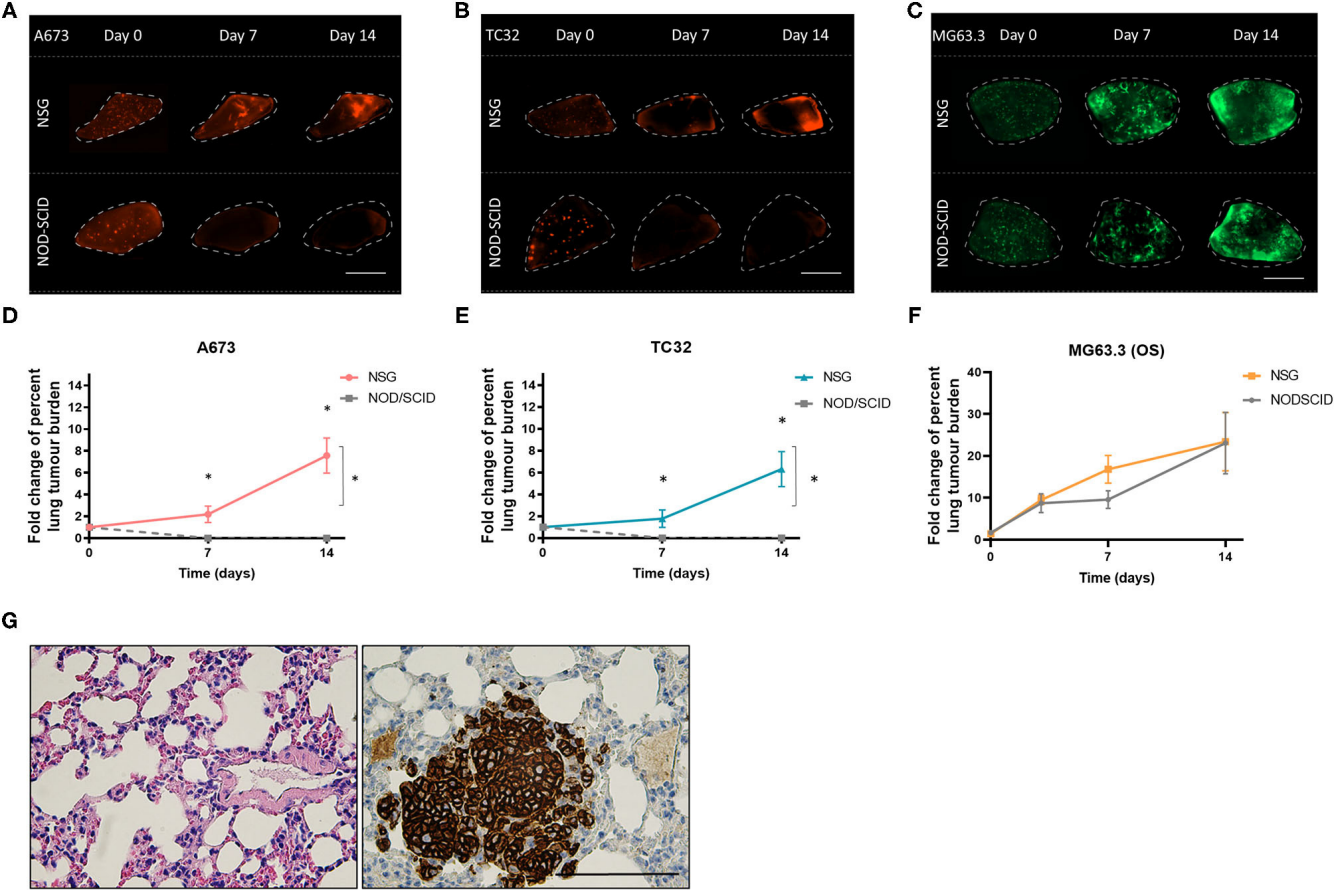
*Ex vivo* growth of EwS cells is maintained in NSG mice. Fluorescence images for A673 **(A)** TC32 **(B)** and MG63.3 **(C)** tdTomato^+^ EwS cells at day 0, 7, and 14 post-injection. Scale bars = 1 mm. Fold change in the percent metastatic tumor burden of A673 **(D)**, TC32 **(E)**, and **(F)** MG63.3 cells. Three animals were included per group (*n* = 3) and a minimum of 10 lung tissue sections were analyzed per animal. Results are shown as mean ± SD. Statistical assessment of differences was determined using two-way ANOVA tests with a Tukey's *post-hoc* test (*p < 0.01). **(G)** CD99 immunoreactive TC32 EwS cells in NSG mouse lung slices at 5 weeks after renal subcapsular implantation of EwS cells. Three animals were included per group (*n* = 3) and a minimum of 10 lung tissue sections were analyzed per animal. Scale bars = 100 μm.

We next assessed the potential impact of residual NK activity in NOD-SCID mice on EwS cell survival in PuMA. To this end, tdTomato-labeled A673 and TC32 EwS cells were tested in PuMA experiments that used NOD-SCID or NSG mice, both carried out using PC medium for subsequent lung explant cultures. To assess lung colonization, the cell surface marker CD99 was used to detect EwS cells by IHC (21). CD99 positive A673 and TC32 cells were confirmed on Day 0 (Figures 1C,D), demonstrating that EwS tumor cells initially arrive in lungs in both strains (average area in μm^2^ = 8 and 18 for NSG and NOD/SCID, respectively). However, by 7 days of *ex vivo* lung culture, CD99 positive cells were only observed in NSG mice (Figures 1C,D). To quantify these findings, tdTomato fluorescence in A673 and TC32 PuMA lung sections were imaged over the same time period. As with CD99 staining, tdTomato-positive cells were observed in both strains at day 0, but only in NSG mice at day 7 and 14 time points of *ex vivo* growth (Figures 2A,B,D,E). Comparatively, MG63.3 OS cells were detected after 14 days in both mouse strains (Figures 2C,F). Lung lesions and microarchitecture observed for EwS cells in PuMA from NSG mice were comparable to *in vivo* results using renal subcapsular injection (Figures 1C,D, 2G). Together, this indicates that EwS cell lung colonization can be assessed using the PuMA model, but that this requires the use of PC medium and NSG mice.

**Figure 3:**
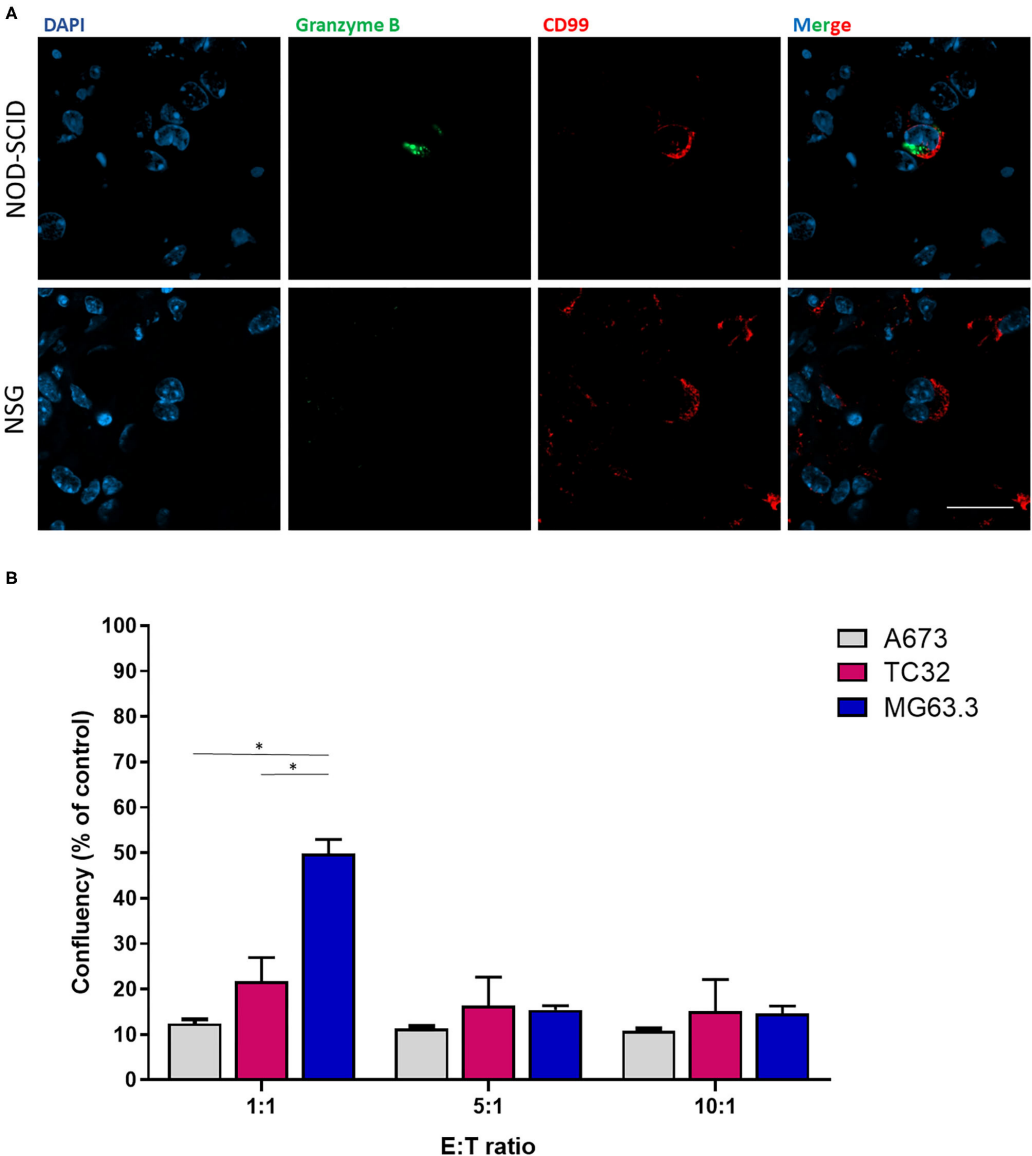
Effects of NK cells on *ex vivo* and *in vitro* EwS cell growth. **(A)** Granzyme B and CD99 co-staining in NOD-SCID and NSG mice at 3-days post-injection of EwS cells. Three animals were included per group (*n* = 3) and a minimum of 10 lung tissue sections were analyzed per animal. **(B)** NK92 cytotoxic effects on EwS compared to OS cells. Ratio-dependent cytotoxicity of NK92 cells was determined for A673, TC32, and MG63.3 cells. Bar graphs represent the mean ± SD of a minimum of three independent experiments. Statistical analysis of differences was calculated using a Student's *t*-test (*p < 0.01).

To examine whether the ability of EwS cells to grow in lung explants of NSG but not NOD-SCID mice was potentially linked to selective NK cytotoxicity in the latter strain, we compared expression of the cytotoxic effector, Granzyme B in lung sections. Granzyme B is a serine protease secreted by cytotoxic T-lymphocytes and NK cells upon binding to target cells, leading to apoptosis of the latter (22). Immunofluorescence of PuMA lung sections from A673 EwS cells revealed the presence of Granzyme B in proximity to CD99-positive cells in NOD-SCID mice, but not NSG mice, at day 3 following tumor cell injection (Figure 3A). To validate that residual NK cells in NOD-SCID lungs might be sufficient to eliminate EwS but not OS cells, the human NK cell line NK92 (effector, E) was co-cultured with EwS or OS cells (tumor, T) at 1:1, 5:1, and 10:1 E:T ratios. After 24 h, the cytotoxic effects of the lowest E:T ratio of 1:1 in both EwS cell lines were significantly enhanced compared to cytotoxicity of MG63.3 cells (*p* = 0.01 and 0.03, respectively), although at higher E:T ratios, OS cells were also killed (Figure 3B).

## Discussion

PuMA has proven to be a very useful *ex vivo* model to study mechanisms of tumor cell lung colonization, particularly in OS (8–10). In a recent study, Morrow et al. document changes in enhancer usage in OS metastatic vs. primary tumor cells driven by the lung microenvironment (12). However, for unknown reasons the PuMA technique has to date not been applicable for EwS cells. In this study we set out to explore possible reasons, including whether the culture conditions and the mouse strains used for conventional PuMA modeling might hinder EwS cell survival. Our results demonstrate the successful adaptation of the PuMA system for studying EwS lung colonization, by substituting B medium for PC medium, and utilizing NSG mice rather than NOD-SCID mice as the host strain.

While NOD-SCID and NSG mouse models both are characterized by T and B cell depletion, loss of C5 complement, and impaired innate immunity, NSG mice are also deficient in NK cells due to the absence of functional receptors for IL-2 and other cytokines (16). Indeed, the beneficial effects of an anti-NK cell antibody before transplantation of human cells in NOD-SCID mice originally led to the development of the NSG model (23), which is associated with improved engraftment of a number of malignant cell types (16–18). Additionally, analysis of immune cell subsets and patient survival revealed that tumor-infiltrating activated NK cells confer prolonged overall survival for EwS patients (24). Previous studies also demonstrated that EwS cells are exquisitely sensitive to NK cell-mediated killing compared with OS and other tumor types (25–27). Our results show Granzyme B secretion in proximity to CD99 positive cells in NOD-SCID mice and significantly enhanced cytotoxic effects of the lowest NK92 E:T ratio of 1:1 in EwS cells compared to OS cells. Adoptive therapy using NK cells overexpressing the activating receptor NKG2D decreased the number of pulmonary metastatic nodules in an EwS NSG xenograft model (28), while in contrast, pharmacological upregulation of NKG2D led to functional NK cells that failed to infiltrate and reduce lung nodules in a nude mouse OS lung metastasis model (29). Together, these data point to NKG2D interactions with its ligands as at least partially explaining enhanced NK cell cytotoxicity in EwS. Accordingly, blocking NKG2D receptor considerably reduced NK cytotoxicity to EwS cells in previous studies (25, 27).

Our results therefore suggest that the immune permissiveness required by EwS cells may result from the lack of active NK cells in NSG mice and additional immune features that should be explored further. Adapting PuMA to NSG mice therefore provides a robust model to study EwS lung metastasis, facilitating the search for novel therapeutically approaches to reduce the burden of metastatic disease in EwS.

## Data Availability Statement

The raw data supporting the conclusions of this article will be made available by the authors, without undue reservation.

## Ethics Statement

The animal study was reviewed and approved by UBC Animal Care Committee (ACC).

## Author Contributions

RS-R conceived the experiments, performed data analysis, and wrote the manuscript. ML and H-FZ performed experiments, performed data analysis, and contributed to the writing and editing of the manuscript. A-CD and CH performed data analysis and contributed to the writing and editing of the manuscript. PS oversaw the project, contributed to the interpretation of results, and co-wrote the manuscript. All authors contributed to the article and approved the submitted version.

## Conflict of Interest

The authors declare that the research was conducted in the absence of any commercial or financial relationships that could be construed as a potential conflict of interest.
